# Gender-Specific Differences on the Association of Hypertension with Subclinical Thyroid Dysfunction

**DOI:** 10.1155/2019/6053068

**Published:** 2019-12-30

**Authors:** Jingkai Zhang, Chao Huang, Zhaowei Meng, Yaguang Fan, Qing Yang, Wenjuan Zhang, Yuxia Gao, Zhenwen Yang, Heng Cai, Bo Bian, Yongle Li, Xuefang Yu, Xin Du, Shaopeng Xu, Jing Nie, Ming Liu, Jinhong Sun, Qing Zhang, Ying Gao, Kun Song, Xing Wang, Li Zhao

**Affiliations:** ^1^Department of Ophthalmology, Tianjin Medical University General Hospital, Tianjin, China; ^2^Hull York Medical School, University of Hull, Hull, UK; ^3^Department of Nuclear Medicine, Tianjin Medical University General Hospital, Tianjin, China; ^4^Tianjin Key Laboratory of Lung Cancer Metastasis and Tumor Microenvironment, Tianjin Lung Cancer Institute, Tianjin Medical University General Hospital, Tianjin, China; ^5^Department of Cardiology, Tianjin Medical University General Hospital, Tianjin, China; ^6^Department of Endocrinology and Metabolism, Tianjin Medical University General Hospital, Tianjin, China; ^7^Department of Health Management, Tianjin Medical University General Hospital, Tianjin, China; ^8^Department of Biochemistry and Molecular Biology, School of Basic Medical Sciences, Tianjin Medical University, Tianjin, China

## Abstract

**Objective:**

Both hypertension and subclinical thyroid dysfunction (STD) have high prevalence and clinical importance, but their relationship is still a matter of debate. We aimed to explore gender-specific difference on the association between hypertension and STD in Chinese.

**Methods:**

We recruited 13,380 ostensible healthy participants (8,237 men and 5,143 women). The associations between hypertension and STD were analyzed on a gender-based setting after dividing STD into subclinical hypothyroidism, subclinical hyperthyroidism and further subgrouped euthyroidism. Crude and adjusted odds ratios of STD for hypertension were analyzed by binary logistic regression.

**Results:**

An increasing trend of hypertension prevalence was found along with aging in both genders. Yet, higher male hypertension prevalence was found until 65 years, and then it intersected with female hypertension prevalence. Women had significantly higher propensity for STD than men. Yet, in elderly participants, this gender-specific difference became less obvious. We displayed detrimental effects for subclinical hypothyroidism in both genders after multiple-covariate adjustments, yet no such effects were shown for subclinical hyperthyroidism. Moreover, females with subclinical hypothyroidism were more likely to be associated with hypertension than males, and the corresponding odds ratios were 1.619 (*P* < 0.01) and 1.557 (*P* < 0.01) and 1.557 (*P* < 0.01) and 1.557 (*P* < 0.01) and 1.557 (*P* < 0.01) and 1.557 (*P* < 0.01) and 1.557 (

**Conclusion:**

We demonstrate that hypertension is associated with subclinical hypothyroidism, but not with subclinical hyperthyroidism. Moreover, females with subclinical hypothyroidism are more likely to be associated with hypertension than males.

## 1. Introduction

Cardiovascular system is recognized as one of the most crucial targets of thyroid hormone. Association of overt thyroid dysfunction and blood pressure is well established [1–3]. Generally, overt hypothyroidism can cause increased diastolic blood pressure [3], whereas overt hyperthyroidism increases pulse pressure [1], respectively. However, the relationship between subclinical thyroid dysfunction (STD) and hypertension is still under debate and has not received sufficient attention. There are studies demonstrating no significant relationship between STD and hypertension [4–6]. But some studies showed an association between subclinical hypothyroidism with hypertension [7–10], while others detected a link between subclinical hyperthyroidism with hypertension [11]. There are also reports showing pregnant women with subclinical hypothyroidism had an increased risk of preeclampsia [12], but levothyroxine treatment could normalize blood pressure without the need of antihypertensive drugs [13]. Besides, another interesting issue is that there seems to be a conflicted gender influence on the association as well. For instance, some investigations identified subclinical hypothyroidism with hypertension only in females [7, 8]. But, Chen et al. [9] demonstrated that male school-aged subjects are more likely to possess this relationship. Yet, Ittermann et al. [10] showed both gender in children and adolescents can have this relationship.

Considering the current uncertainty and inconformity regarding STD and hypertension, the purpose of this observational research was to investigate this association systematically in a cohort of Chinese with the largest sample size so far, and special attention was paid on the gender differences on the relationship.

## 2. Methods

### 2.1. Population and Data Acquisition

A health-checking survey has been conducted in our institute for well over a decade, which was a collaborative investigation from a number of departments. The method was described in detail as previously reported [14–26]. In brief, the self-reported healthy participants completed the questionnaire, underwent a physical examination, and provided their blood samples. For the current analytical purpose, individuals with known thyroid, heart, renal, hepatic, oncologic, infectious or immune diseases or current pregnancy or taking contraceptive drugs were excluded. Subjects with overt thyroid disorders or taking any drugs that might influence thyroid function were ruled out of the current investigation. Similarly, subjects with hypertension or hypertensive patients receiving treatment were also excluded from this research. All the ostensible healthy subjects were included in the current analysis. As a result, a total number of 13,380 eligible subjects (8237 men and 5143 women) with adequate data for analysis were included. Tianjin Medical University General Hospital review board and ethic committee approved this investigation. Written informed consent was obtained from all the participants before data collection.

### 2.2. Measurements

All participants were interviewed to obtain a complete medical history, as well as detailed sociodemographic characteristics, family history, and menstruation pattern in women. And then, anthropometric measurements were conducted according to the following method. After seated for at least 10 minutes, a qualified physician measured blood pressure with a standard Mercury sphygmomanometer three times at a minimum interval of 1 minute. And, the mean value was recorded as the subjects' final blood pressure. Body mass index (BMI) was calculated as weight (kilograms)/height^2^ (meters^2^). Weight was examined in light indoor clothing without shoes to the nearest 100 gram. Height was measured without shoes to the nearest 1 centimeter with a stadiometer.

Fasting blood samples were obtained in the same day and then processed and tested in a central clinical laboratory in our hospital. The blood parameters used in this study included the following items: thyroid stimulating hormone (TSH) also called thyrotropin, free tri-iodothyronine (FT3), free thyroxine (FT4), total cholesterol (TC), triglycerides (TG), alanine aminotransferase (ALT), aspartate transaminase (AST), total bilirubin (TBIL), blood urea nitrogen (BUN), creatinine (Cr), and fasting glucose (FG). Thyroid function indices (TSH, FT3, and FT4) were determined by chemiluminescence immunoassay reaction principle on an automated ADVIA Centaur analyzer (Siemens Healthcare Diagnostics, Erlangen, Germany). The reference ranges for blood parameters were as follows: TSH 0.3–5.0 mIU/L, FT3 3.5–6.5 pmol/L, FT4 11.5–23.5 pmol/L, TC 3.59–5.18 mmol/L, TG 0.57–1.70 mmol/L, ALT 5–40 U/L, AST 5–40 U/L, TBIL 3.4–20 *μ*mol/L, BUN 1.7–8.3 mmol/L, Cr 44–115 *μ*mol/L, and FG 3.6–5.8 mmol/L.

### 2.3. Definition

Systolic blood pressure (SBP) of ≥140 mmHg and diastolic blood pressure (DBP) ≥90 mmHg, respectively, were considered increased, and hypertension was defined as increased SBP and/or increased DBP [27, 28]. Thyroid function subgroups were determined according to the TSH level since overt thyroid dysfunction cases were excluded from the study. Subclinical hypothyroidism was defined as TSH >5.0 mIU/L, subclinical hyperthyroidism as TSH ≤0.3 mIU/L, and the rest as euthyroidism. Moreover, in order to further stratify participants for easier logistic regression analyses, euthyroidism was subgrouped according to the TSH values: 0.3 *μ*lU/mL < TSH ≤ 1.0 *μ*lU/mL, 1.0 *μ*lU/mL < TSH ≤ 2.0 *μ*lU/mL, 2.0 *μ*lU/mL < TSH ≤ 3.0 *μ*lU/mL, 3.0 *μ*lU/mL < TSH ≤ 4.0 *μ*lU/mL, 4.0 *μ*lU/mL < TSH ≤ 5.0 *μ*lU/mL. Age subgroups were determined as the below cutoff thresholds: age ≤25 years, 25 years < age ≤ 35 years, 35 years < age ≤ 45 years, 45 years < age ≤ 55 years, 55 years < age ≤ 65 years, 65 years < age ≤ 75 years, and age >75 years.

### 2.4. Statistical Analysis

Statistical analyses were performed by the software of Statistical Package for Social Sciences (SPSS version 23.0, Chicago, IL, USA). Continuous data with quantitative characteristics were presented as mean ± standard deviation, while categorical data with qualitative characteristics as numbers and percentages. Intergroup value differences were analyzed by independent sample's *t*-test, while intergroup prevalence differences by Chi-squared test. Logistic regression was employed to assess the association between TSH levels and hypertension as well as increased DBP/SBP, where both crude and adjusted odds ratios were reported together with 95% confidence intervals (CI). *P* < 0.05 was considered as statistically significant.

## 3. Results

### 3.1. General Characteristics of the Participants

**Figure 1:**
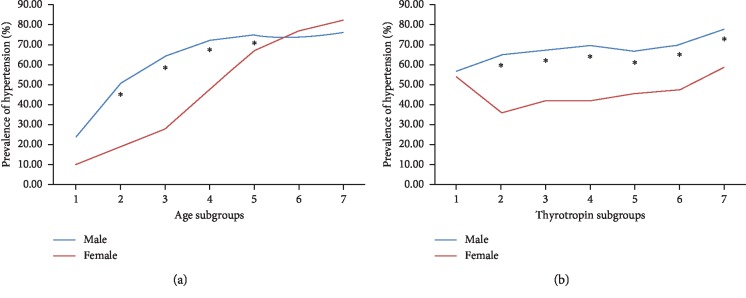
Prevalence of hypertension in different age subgroups or different thyrotropin subgroups. (a) Age subgroups 1 to 7 are referred as follows: age < 25 years, 25 years ≤ age < 35 years, 35 years ≤ age < 45 years, 45 years ≤ age < 55 years, 55 years ≤ age < 65 years, 65 years < age ≤ 75 years, and age >75 years. ^*∗*^Difference of prevalence between gender was significant at 0.01. (b) Thyrotropin subgroups 1 to 7 are referred as follows: TSH ≤ 0.3 mIU/L, 0.3 *μ*lU/mL < TSH ≤ 1.0 *μ*lU/mL, 1.0 *μ*lU/mL < TSH ≤ 2.0 *μ*lU/mL, 2.0 *μ*lU/mL < TSH ≤ 3.0 *μ*lU/mL, 3.0 *μ*lU/mL < TSH ≤ 4.0 *μ*lU/mL, 4.0 *μ*lU/mL < TSH ≤ 5.0 *μ*lU/mL, TSH > 5.0 mIU/L. ^*∗*^Difference of prevalence between gender was significant at 0.01.

**Figure 2:**
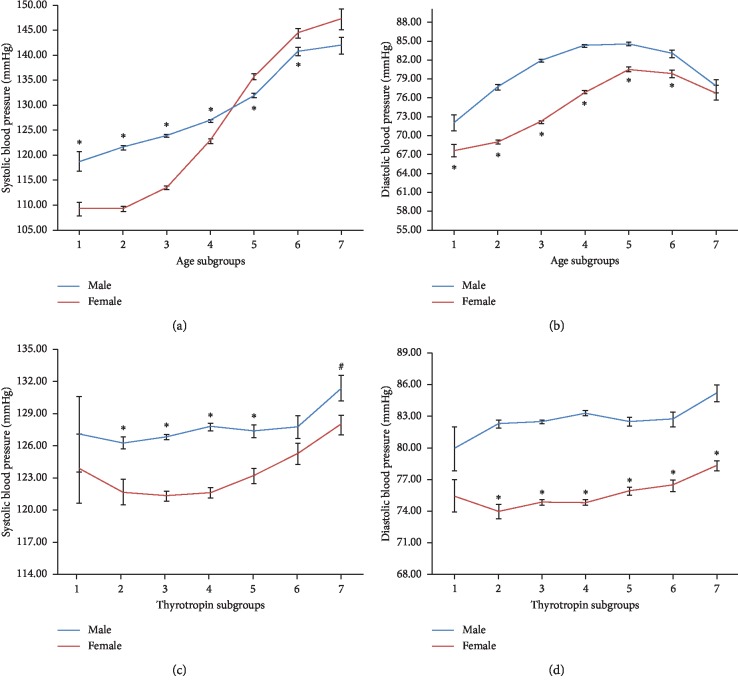
Systolic blood pressure and diastolic blood pressure levels in different age subgroups or different thyrotropin subgroups. (a, b) Age subgroups 1 to 7 are referred as follows: age < 25 years, 25 years ≤ age < 35 years, 35 years ≤ age < 45 years, 45 years ≤ age < 55 years, 55 years ≤ age < 65 years, 65 years < age ≤ 75 years, and age >75 years. ^*∗*^Difference of blood pressure levels between genders was significant at 0.01. (c, d) Thyrotropin subgroups 1 to 7 are referred as follows: TSH ≤ 0.3 mIU/L, 0.3 *μ*lU/mL < TSH ≤ 1.0 *μ*lU/mL, 1.0 *μ*lU/mL < TSH ≤ 2.0 *μ*lU/mL, 2.0 *μ*lU/mL < TSH ≤ 3.0 *μ*lU/mL, 3.0 *μ*lU/mL < TSH ≤ 4.0 *μ*lU/mL, 4.0 *μ*lU/mL < TSH ≤ 5.0 *μ*lU/mL, and TSH > 5.0 mIU/L. ^#^Difference of blood pressure levels between genders was significant at 0.05; ^*∗*^Difference of blood pressure levels between genders was significant at 0.01.

The clinical characteristics of each gender are summarized and displayed in total as well as by gender in Table 1. Gender differences were identified in some clinical parameters. For instance, males had larger BMI than females. Men showed lower TSH but higher FT3 and FT4 than women. A general increasing trend of hypertension prevalence was found along with aging in both genders. Yet, higher male hypertension prevalence was found from the age of 25 years until 65 years, and then an intersecting pattern appeared, indicating that elderly women (after the period of menopause) would have the same or even higher hypertension trait than men (Figure 1(a)). As for different TSH levels, men were discovered to have higher hypertension prevalence than women with the exception of the lowest TSH subgroup (Figure 1(b)). If SBP and DBP were analyzed separately, the age-related intersecting pattern was even more obvious in SBP. Elderly women showed significantly higher SBP level than elderly men (Figure 2(a)). But regarding DBP level, after reaching a plateau around the age of 65 years, DBP demonstrated a decreasing trend in elderly people for both genders (Figure 2(b)). The gender differences of SBP and DBP according to different TSH levels were roughly the same as the male dominant hypertension prevalence pattern (Figures 2(c) and 2(d)).

### 3.2. Incidence of Subclinical Thyroid Dysfunction according to Age Subgroups

Females showed significantly higher overall incidence of subclinical hypothyroidism (9.62%) and subclinical hyperthyroidism (0.80%) than males (2.85% and 0.25%, respectively). If detailed incidences were analyzed according to age subgroups, the youngest subgroup demonstrated the same female dominant pattern of differences (Table 2).

### 3.3. Hypertension in Different TSH Levels

For the aim of assessing different associations between thyroid functions and hypertension, we used binary logistic regression for calculations with the middle level of TSH (2.0 *μ*lU/mL to 3.0 *μ*lU/mL) as reference (Table 3). We only displayed negative effects for subclinical hypothyroidism in both genders after multiple-covariate adjustments, yet no such effects were shown for subclinical hyperthyroidism. Moreover, the adjusted ORs of hypertension in participants with subclinical hypothyroidism were much higher and more statistically significant in females than in males, and the corresponding ORs were 1.557 (*P* < 0.05) and 1.619 (*P* < 0.01) for men and women, respectively.

### 3.4. Increased SBP and DBP in Different TSH Levels

Next, we assessed the association between TSH versus SBP and DBP separately by using binary logistic regression (Table 4). In conformity with the findings in Table 3, there were at least two important results in this analytical setting. First, adjusted detrimental effects for increased SBP and DBP were both displayed for subclinical hypothyroidism, not for subclinical hyperthyroidism. Second, women were found to have higher adjusted ORs than males for increased SBP and DBP. For example, the adjusted ORs for SBP were 1.371 (*P* < 0.05) for women, but 1.154 (*P* > 0.05) for men. And, for DBP, the corresponding ORs were 1.604 (*P* < 0.01) for women, and 1.564 (*P* < 0.01) for men.

## 4. Discussion

Normal thyroid function is essential because thyroid hormones affect a multitude of biological processes in the body, including its well-recognized effects on cardiovascular system and blood pressure. In fact, blood pressure is altered across the entire spectrum of thyroid dysfunctions [1]. The effects of hypothyroidism include decreased cardiac output, narrow pulse pressure, increased systemic vascular resistance, and decreased metabolic rate. The manifestations of hyperthyroidism are in marked contrast to those of hypothyroidism and include increased cardiac output, contractility, tachycardia, widened pulse pressure, decreased systemic vascular resistance, and increased basal metabolic rate. Yet, the relationship between STD and hypertension has remained controversial and insufficiently studied. The current investigation was set to perform a comprehensive statistical analysis on the largest cross-sectional population so far to resolve the issue. We have two major findings. First, hypertension is associated with subclinical hypothyroidism, but not with subclinical hyperthyroidism. Second, gender-specific difference exists, and females with subclinical hypothyroidism are more likely to develop hypertension than males.

In fact, very similar to the relationship of overt hypothyroidism and blood pressure, there is much more solid evidence reporting why subclinical hypothyroidism is associated with hypertension, but not subclinical hyperthyroidism. Cai et al. [29] organized a meta-analysis in 2011, retrieving articles that addressed the association between STD and hypertension. This meta-analysis indicates that only subclinical hypothyroidism is associated with increased SBP and DBP, whereas subclinical hyperthyroidism is not. A recent publication by He et al. [30] conducted another meta-analysis showing a dose-response relationship between serum TSH level and hypertension risk even in euthyroid individuals, suggesting individuals with higher normal TSH level are at higher risk of developing hypertension than those with lower normal TSH level. Reports from Japan [5] and Germany [6, 31] demonstrated this phenomenon existed despite countries of different iodine supply in food (iodine-replete in Japan while iodine-deficient in Germany) as well as prevalence of hypertension (higher in Japan than Germany). There are several putative mechanisms regarding this clinical finding [32]. Endothelial dysfunction is very obvious in subclinical hypothyroidism and is attributed to a reversible defect in the production of NO [33]. Acceleration of arterial stiffness [34, 35] and increased renal vascular resistance [36, 37] are also related with high serum TSH level. Subclinical dysfunction is also associated with impaired left ventricular function and left ventricular systolic dysfunction [38]. Increased carotid artery intima-media thickness has also been reported in subclinical hypothyroidism, which is reversible after normalization of TSH levels [39]. And finally, possible independent effects of TSH itself on the cardiovascular system through acting on its receptor directly should also be considered. TSH receptors have been shown to exist on coronary artery smooth muscle cells [40] and the heart muscle [41]. Many of the above mechanisms happens directly because of relatively high TSH concentration in subclinical hypothyroidism; therefore, subclinical hyperthyroidism could not have such effects.

Sex dimorphism exists in both thyroid disorders and hypertension. We found women had significantly higher propensity for subclinical hypothyroidism and subclinical hyperthyroidism than men. Yet, for elderly participants (after the period of menopause), the gender-specific difference became less obvious (Table 2). From our understanding, effects of estrogens on developing thyroid disorders gradually diminish; as people grow old, ovarian function to produce estrogens will subside. Perhaps, this could be a crucial underpinning mechanism. However, on the contrary, higher male hypertension prevalence was found from the age of 25 years until 65 years, and then an intersecting pattern appeared, indicating that after the period of menopause, with the declining protection from circulation estrogens, women would have the same or even higher hypertension prevalence (Figure 1). The focus of the current study is the net effect of gender on thyroid disorders and hypertension, which means females with subclinical hypothyroidism are more likely to develop hypertension than males. We consider that it is possibly due to the estrogen's protective effect on hypertension is overshadowed and overwhelmed by the female inclination of thyroid dysfunction. The same phenomenon is reported in our previous study on the relationship between thyroid dysfunction and metabolic syndrome, which showed women with high TSH were more likely to have metabolic syndrome [16]. Besides sex hormone influence, there could be some lifestyle differences in men than in women that would be more easily missed during the basic information acquirement and questionnaire collection. Men would be more prone to smoke or drink or have bad eating habits as previously indicated [7, 10], and this bias would ensue a negative result in men.

There are several limitations of our study. First, although this is the biggest observational study on this subject so far, a disadvantage arises from the cross-sectional design, which allows only limited validity according to causality. Well-planned prospective cohort study should be conducted in the future. Besides, outcome-based studies (e.g., long-term mortality, stroke, and myocardial infarction from the same cohort) should be planned to see if TSH, sex, and hypertension are in any way associated with harder outcomes. And, we also suggest a trial to be considered looking at whether therapy for subclinical hypothyroidism (e.g., levothyroxine replacement) corrects the hypertension differences. Second, we did not measure sex hormones, thyroid antibodies, and iodine levels in our population, we neither double checked blood parameters because there is a relative budget limitation. Third, some participants could possibly avoid telling us their bad lifestyle habits (such as drinking, smoking or high-calorie food eating) tentatively, which could influence our results. Fourth, although we applied strict exclusion criteria to rule our influencing factors, a number of the participants with various diseases might not be aware of their medical conditions, which could also became a confounding factor in our study. Finally, the number of cases with subclinical hyperthyroidism is relatively small in the current study; future study should focus on this subgroup of people in purpose.

In conclusion, we demonstrate that hypertension is associated with subclinical hypothyroidism, but not with subclinical hyperthyroidism. Moreover, a gender-specific pattern exists, and females with subclinical hypothyroidism are more likely to be associated with hypertension than males.

## Figures and Tables

**Table 1:** Participant characteristics based on different genders.

	Total	Male	Female	*T* value
Case number	13380	8237	5143	
Age (years)	48.63 ± 10.69	48.55 ± 10.37	48.76 ± 11.18	−1.12
BMI (kg/m^2^)	25.53 ± 3.45	26.27 ± 3.21	24.33 ± 3.47	32.99^*∗∗*^
TSH (*μ*lU/mL)	2.40 ± 2.09	2.12 ± 2.01	2.84 ± 2.15	−19.50^*∗∗*^
FT3 (pmol/L)	5.20 ± 0.52	5.39 ± 0.46	4.90 ± 0.45	59.68^*∗∗*^
FT4 (pmol/L)	16.22 ± 2.08	16.69 ± 2.06	15.47 ± 1.89	34.32^*∗∗*^
TC (mmol/L)	5.15 ± 0.99	5.13 ± 0.96	5.18 ± 1.03	−2.84^*∗∗*^
TG (mmol/L)	1.74 ± 1.34	1.98 ± 1.47	1.35 ± 0.99	26.89^*∗∗*^
ALT (U/L)	22.65 ± 14.01	26.05 ± 14.76	17.20 ± 10.67	37.36^*∗∗*^
AST (U/L)	19.69 ± 7.48	20.62 ± 7.72	18.19 ± 6.80	18.56^*∗∗*^
TBIL (*μ*mol/L)	11.89 ± 5.11	13.02 ± 5.32	10.09 ± 4.19	33.62^*∗∗*^
BUN (mmol/L)	4.71 ± 1.33	4.94 ± 1.30	4.34 ± 1.29	26.06^*∗∗*^
Cr (*μ*mol/L)	73.46 ± 14.71	81.07 ± 11.71	61.27 ± 10.10	100.21^*∗∗*^
FG (mmol/L)	5.42 ± 1.20	5.56 ± 1.29	5.20 ± 1.00	17.08^*∗∗*^

BMI = body mass index; TSH = thyroid stimulation hormone; FT3 = free tri-iodothyronine; FT4 = free thyroxine; TC = total cholesterol; TG = triglycerides; ALT = alanine aminotransferase; AST = aspartate transaminase; TBIL = total bilirubin; BUN = blood urea nitrogen; Cr = creatinine; FG = fasting glucose. ^*∗∗*^*P* < 0.01 (analyzed by independent sample's *t*-test).

**Table 2:** Incidence of subclinical hypothyroidism and subclinical hyperthyroidism on different genders according to age.

	Incidence (and case number count) in different age subgroups (years)							
	Age ≤ 25	25 < age ≤ 35	35 < age ≤ 45	45 < age ≤ 55	55 < age ≤ 65	65 < age ≤ 75	Age > 75	Total
*Male*								
Euthyroidism	97.83% (45)	97.53% (670)	97.71% (2641)	97.43% (2690)	96.03% (1500)	91.60% (338)	88.99% (97)	96.89% (7981)
Subclinical hypothyroidism^#^	2.17% (1)	2.04% (14)	2.11% (57)	2.39% (66)	3.52% (55)	8.40% (31)	10.09% (11)	2.85% (235)
Subclinical hyperthyroidism^#^	0.00% (0)	0.44% (3)	0.18% (5)	0.18% (5)	0.45% (7)	0.00% (0)	0.92% (1)	0.25% (21)

*Female*								
Euthyroidism	90.00% (45)	94.49% (480)	92.18% (1474)	89.25% (1428)	84.77% (824)	85.26% (295)	89.71% (61)	89.58% (4607)
Subclinical hypothyroidism^#^	10.00% (5)	5.51% (28)	7.19% (115)	9.81% (157)	13.99% (136)	13.87% (48)	8.82% (6)	9.62% (495)
Subclinical hyperthyroidism^#^	0.00% (0)	0.00% (0)	0.63% (10)	0.94% (15)	1.23% (12)	0.87% (3)	1.47% (1)	0.80% (41)

*Chi-squared value * ^*^*^								
Subclinical hypothyroidism^#^	2.504	10.296^*∗∗*^	68.193^*∗∗*^	116.481^*∗∗*^	95.568^*∗∗*^	5.637^*∗*^	0.71	284.382^*∗∗*^
Subclinical hyperthyroidism^#^	—	2.145	6.177^*∗*^	14.249^*∗∗*^	6.302^*∗*^	3.419	0.108	23.148^*∗∗*^
Total	2.504	12.536^*∗∗*^	73.723^*∗∗*^	128.940^*∗∗*^	100.369^*∗∗*^	8.849^*∗*^	0.186	304.206^*∗∗*^

^#^Subclinical hypothyroidism defined as TSH > 5.0 *μ*lU/mL, subclinical hyperthyroidism defined as TSH ≤ 0.3 *μ*lU/mL. ^^^Comparing the incidence of subclinical hypothyroidism and/or subclinical hyperthyroidism between males and females by Chi-squared method. ^*∗*^*P* < 0.05 and ^*∗∗*^*P* < 0.01.

**Table 3:** Hypertension in different genders according to thyroid functions.

	Males		Females	
Thyroid functions^#^	Crude OR (CI)	Adjusted OR (CI)^#^	Crude OR (CI)	Adjusted OR (CI)^#^
Subclinical hyperthyroidism TSH ≤ 0.3 *μ*lU/mL	0.577 (0.242–1.376)	0.757 (0.301–1.906)	1.603 (0.861–2.988)	1.357 (0.677–2.719)
0.3 *μ*lU/mL < TSH≤1.0 *μ*lU/mL	0.811 (0.684–0.961)^*∗*^	0.833 (0.695–1.001)	0.784 (0.605–1.015)	0.731 (0.542–1.001)
1.0 *μ*lU/mL < TSH≤2.0 *μ*lU/mL	0.899 (0.803–1.007)	0.930 (0.825–1.049)	1.010 (0.876–1.164)	1.103 (0.937–1.297)
2.0 *μ*lU/mL < TSH≤3.0 *μ*lU/mL	Reference	Reference	Reference	Reference
3.0 *μ*lU/mL < TSH≤4.0 *μ*lU/mL	0.881 (0.739–1.051)	0.816 (0.676–1.001)	1.164 (0.977–1.387)	1.117 (0.916–1.363)
4.0 *μ*lU/mL < TSH≤5.0 *μ*lU/mL	1.015 (0.774–1.331)	1.044 (0.781–1.397)	1.243 (1.002–1.543)^*∗*^	1.026 (0.801–1.313)
Subclinical hypothyroidism TSH>5.0 *μ*lU/mL	1.523 (1.105–2.100)^*∗*^	1.557 (1.105–2.192)^*∗*^	1.959 (1.594–2.407)^*∗∗*^	1.619 (1.282–2.045)^*∗∗*^

TSH = thyroid stimulation hormone; OR = odds ratio; CI = confidence interval. ^#^Logistic regression model including age, body mass index, total cholesterol, triglycerides, alanine aminotransferase, aspartate transaminase, total bilirubin, blood urea nitrogen, creatinine, and fasting glucose as covariates. ^*∗*^*P* < 0.05 and ^*∗∗*^*P* < 0.01.

**Table 4:** Increased SBP and DBP in different genders according to thyroid functions.

	Males SBP		Males DBP		Females SBP		Females DBP	
Thyroid functions^#^	Crude OR (CI)	Adjusted OR (CI)^#^	Crude OR (CI)	Adjusted OR (CI)^#^	Crude OR (CI)	Adjusted OR (CI)^#^	Crude OR (CI)	Adjusted OR (CI)^#^
Subclinical hyperthyroidism TSH ≤ 0.3 *μ*lU/mL	0.948 (0.346–2.601)	1.285 (0.432–3.828)	0.588 (0.247–1.403)	0.796 (0.317–1.998)	1.648 (0.816–3.329)	1.258 (0.537–2.948)	1.453 (0.781–2.703)	1.284 (0.653–2.524)
0.3 *μ*lU/mL < TSH ≤ 1.0 *μ*lU/mL	0.906 (0.750–1.095)	0.969 (0.789–1.189)	0.823 (0.694–0.975)^*∗*^	0.847 (0.707–1.014)	1.315 (0.972–1.779)	1.323 (0.903–1.938)	0.755 (0.580–0.982)^*∗*^	0.733 (0.547–1.001)
1.0 *μ*lU/mL < TSH ≤ 2.0 *μ*lU/mL	0.845 (0.747–1.001)	0.894 (0.782–1.022)	0.892 (0.797–1.001)	0.919 (0.816–1.036)	1.012 (0.844–1.213)	1.138 (0.912–1.420)	1.002 (0.868–1.156)	1.085 (0.926–1.271)
2.0 *μ*lU/mL < TSH ≤ 3.0 *μ*lU/mL	Reference	Reference	Reference	Reference	Reference	Reference	Reference	Reference
3.0 *μ*lU/mL < TSH ≤ 4.0 *μ*lU/mL	0.887 (0.730–1.078)	0.788 (0.637–1.001)	0.847 (0.711–1.009)	0.788 (0.654–1.001)	1.291 (1.041–1.600)^*∗*^	1.333 (1.028–1.729)^*∗*^	1.153 (0.966–1.376)	1.107 (0.911–1.345)
4.0 *μ*lU/mL < TSH ≤ 5.0 *μ*lU/mL	1.125 (0.850–1.489)	1.072 (0.788–1.460)	0.952 (0.729–1.243)	0.978 (0.736–1.301)	1.579 (1.226–2.033)^*∗∗*^	1.326 (1.001–1.805)^*∗*^	1.270 (1.022–1.577)^*∗*^	1. 081 (0.850–1.375)
Subclinical hypothyroidism TSH > 5.0 *μ*lU/mL	1.451 (1.085–1.939)^*∗*^	1.154 (0.835–1.595)	1.479 (1.077–2.031)^*∗*^	1.564 (1.117–2.190)^*∗∗*^	1.862 (1.474–2.352)^*∗∗*^	1.371 (1.032–1.823)^*∗*^	1.891 (1.540–2.322)^*∗∗*^	1.604 (1.279–2.012)^*∗∗*^

TSH = thyroid stimulation hormone; SBP = systolic blood pressure; DBP = diastolic blood pressure; OR = odds ratio; CI = confidence interval. ^#^Logistic regression model including age, body mass index, total cholesterol, triglycerides, alanine aminotransferase, aspartate transaminase, total bilirubin, blood urea nitrogen, creatinine, and fasting glucose as covariates. ^*∗*^*P* < 0.05 and ^*∗∗*^*P* < 0.01.

## Data Availability

The data used to support the findings of this study are available from the corresponding author upon request.

## References

[1] Danzi S., Klein I. (2003). Thyroid hormone and blood pressure regulation. *Current Hypertension Reports*.

[2] Klein I., Ojamaa K. (2001). Thyroid hormone and the cardiovascular system. *New England Journal of Medicine*.

[3] Prisant L. M., Gujral J. S., Mulloy A. L. (2006). Hyperthyroidism: a secondary cause of isolated systolic hypertension. *The Journal of Clinical Hypertension*.

[4] Duan Y., Peng W., Wang X. (2009). Community-based study of the association of subclinical thyroid dysfunction with blood pressure. *Endocrine*.

[5] Takashima N., Niwa Y., Mannami T., Tomoike H., Iwai N. (2007). Characterization of subclinical thyroid dysfunction from cardiovascular and metabolic viewpoints: the suita study. *Circulation Journal*.

[6] Völzke H., Ittermann T., Schmidt C. O. (2009). Subclinical hyperthyroidism and blood pressure in a population-based prospective cohort study. *European Journal of Endocrinology*.

[7] Duan Y., Wang X., Peng W. (2009). Gender-specific associations between subclinical hypothyroidism and blood pressure in Chinese adults. *Endocrine*.

[8] Jian W., Jin J., Qin L. (2013). Relationship between thyroid-stimulating hormone and blood pressure in the middle-aged and elderly population. *Singapore Medical Journal*.

[9] Chen H., Xi Q., Zhang H. (2012). Investigation of thyroid function and blood pressure in school-aged subjects without overt thyroid disease. *Endocrine*.

[10] Ittermann T., Thamm M., Wallaschofski H., Rettig R., Völzke H. (2012). Serum thyroid-stimulating hormone levels are associated with blood pressure in children and adolescents. *The Journal of Clinical Endocrinology & Metabolism*.

[11] Walsh J. P., Bremner A. P., Bulsara M. K. (2006). Subclinical thyroid dysfunction and blood pressure: a community-based study. *Clinical Endocrinology*.

[12] Wilson K. L., Casey B. M., McIntire D. D., Halvorson L. M., Cunningham F. G. (2012). Subclinical thyroid disease and the incidence of hypertension in pregnancy. *Obstetrics & Gynecology*.

[13] Ramtahal R., Dhanoo A. (2016). Subclinical hypothyroidism causing hypertension in pregnancy. *Journal of the American Society of Hypertension*.

[14] Liu L., Lou S., Xu K., Meng Z., Zhang Q., Song K. (2013). Relationship between lifestyle choices and hyperuricemia in Chinese men and women. *Clinical Rheumatology*.

[15] Zhang Q., Lou S., Meng Z., Ren X. (2011). Gender and age impacts on the correlations between hyperuricemia and metabolic syndrome in Chinese. *Clinical Rheumatology*.

[16] Meng Z., Liu M., Zhang Q. (2015). Gender and age impact on the association between thyroid-stimulating hormone and serum lipids. *Medicine*.

[17] Meng Z., Liu M., Zhang Q. (2015). Gender and age impacts on the association between thyroid function and metabolic syndrome in Chinese. *Medicine*.

[18] Ren X., Meng Z., Liu M. (2016). No associations exist between mean platelet volume or platelet distribution width and thyroid function in Chinese. *Medicine*.

[19] Wang S., Zhang J., Zhu L. (2017). Association between liver function and metabolic syndrome in Chinese men and women. *Scientific Reports*.

[20] Zhang J., Meng Z., Zhang Q. (2016). Gender impact on the correlations between subclinical thyroid dysfunction and hyperuricemia in Chinese. *Clinical Rheumatology*.

[21] Zhou P., Meng Z., Liu M. (2016). The associations between leukocyte, erythrocyte or platelet, and metabolic syndrome in different genders of Chinese. *Medicine*.

[22] Liu X., Zhang C., Meng Z. (2018). Waist circumference and subclinical thyroid dysfunction in a large cohort of Chinese men and women. *Endocrine Practice*.

[23] Zhang C., Meng Z., Li X. (2018). No associations exists between red blood cell distribution width and serum uric acid in both sexes. *Medicine*.

[24] Zhang X., Meng Z., Li X. (2018). The association between total bilirubin and serum triglyceride in both sexes in Chinese. *Lipids in Health and Disease*.

[25] Liu X., Zhang J., Meng Z. (2018). Gender impact on the correlations between Graves’ hyperthyroidism and hyperuricemia in Chinese. *Irish Journal of Medical Science (1971)*.

[26] Zhao F., Yan Z., Meng Z. (2018). Relationship between mean platelet volume and metabolic syndrome in Chinese patients. *Scientific Reports*.

[27] Chobanian A. V., Bakris G. L., Black H. R. (2003). The seventh report of the joint national committee on prevention, detection, evaluation, and treatment of high blood pressure: the JNC 7 report. *JAMA*.

[28] Mancia G., De Backer G., Dominiczak A. (2007). 2007 guidelines for the management of arterial hypertension: the task force for the management of arterial hypertension of the European society of hypertension (ESH) and of the European society of cardiology (ESC). *Journal of Hypertension*.

[29] Cai Y., Ren Y., Shi J. (2011). Blood pressure levels in patients with subclinical thyroid dysfunction: a meta-analysis of cross-sectional data. *Hypertension Research: Official Journal of the Japanese Society of Hypertension*.

[30] He W., Li S., Wang B. (2019). Dose-response relationship between thyroid stimulating hormone and hypertension risk in euthyroid individuals. *Journal of Hypertension*.

[31] Volzke H., Alte D., Dorr M. (2006). The association between subclinical hyperthyroidism and blood pressure in a population-based study. *Journal of Hypertension*.

[32] Stabouli S., Papakatsika S., Kotsis V. (2010). Hypothyroidism and hypertension. *Expert Review of Cardiovascular Therapy*.

[33] Taddei S., Caraccio N., Virdis A. (2003). Impaired endothelium-dependent vasodilatation in subclinical hypothyroidism: beneficial effect of levothyroxine therapy. *The Journal of Clinical Endocrinology and Metabolism*.

[34] Nagasaki T., Inaba M., Kumeda Y. (2006). Increased pulse wave velocity in subclinical hypothyroidism. *The Journal of Clinical Endocrinology and Metabolism*.

[35] Lambrinoudaki I., Armeni E., Rizos D. (2012). High normal thyroid-stimulating hormone is associated with arterial stiffness in healthy postmenopausal women. *Journal of Hypertension*.

[36] Gumieniak O., Perlstein T. S., Hopkins P. N. (2004). Thyroid function and blood pressure homeostasis in euthyroid subjects. *The Journal of Clinical Endocrinology and Metabolism*.

[37] Tsuda A., Inaba M., Ichii M. (2013). Relationship between serum TSH levels and intrarenal hemodynamic parameters in euthyroid subjects. *European Journal of Endocrinology*.

[38] Biondi B. (2007). Cardiovascular effects of mild hypothyroidism. *Thyroid: Official Journal of the American Thyroid Association*.

[39] Kim S. K., Kim S. H., Park K. S., Park S. W., Cho Y. W. (2009). Regression of the increased common carotid artery-intima media thickness in subclinical hypothyroidism after thyroid hormone replacement. *Endocrine Journal*.

[40] Sellitti D. F., Dennison D., Akamizu T., Doi S. Q., Kohn L. D., Koshiyama H. (2000). Thyrotropin regulation of cyclic adenosine monophosphate production in human coronary artery smooth muscle cells. *Thyroid: Official Journal of the American Thyroid Association*.

[41] Drvota V., Janson A., Norman C. (1995). Evidence for the presence of functional thyrotropin receptor in cardiac muscle. *Biochemical and Biophysical Research Communications*.

